# A Phylogenomic Backbone for Acoelomorpha Inferred From Transcriptomic Data

**DOI:** 10.1093/sysbio/syae057

**Published:** 2024-10-25

**Authors:** Samuel Abalde, Ulf Jondelius

**Affiliations:** Department of Zoology, Swedish Museum of Natural History, Stockholm, Sweden; Department of Zoology, Swedish Museum of Natural History, Stockholm, Sweden

**Keywords:** Acoelomorpha, ancestral character state reconstruction, divergence times, incomplete lineage sorting, introgression, morphological phylogenetics, transcriptomics

## Abstract

Xenacoelomorpha are mostly microscopic, morphologically simple worms, lacking many structures typical of other bilaterians. Xenacoelomorphs—which include three main groups, namely Acoela, Nemertodermatida, and *Xenoturbella*—have been proposed to be an early diverging Bilateria, sister to protostomes and deuterostomes, but other phylogenomic analyses have recovered this clade nested within the deuterostomes, as sister to Ambulacraria. The position of Xenacoelomorpha within the metazoan tree has understandably attracted a lot of attention, overshadowing the study of phylogenetic relationships within this group. Given that *Xenoturbella* includes only six species whose relationships are well understood, we decided to focus on the most speciose Acoelomorpha (Acoela + Nemertodermatida). Here, we have sequenced 29 transcriptomes, doubling the number of sequenced species, to infer a backbone tree for Acoelomorpha based on genomic data. The recovered topology is mostly congruent with previous studies. The most important difference is the recovery of *Paratomella* as the first off-shoot within Acoela, dramatically changing the reconstruction of the ancestral acoel. Besides, we have detected incongruence between the gene trees and the species tree, likely linked to incomplete lineage sorting, and some signal of introgression between the families Dakuidae and Mecynostomidae, which hampers inferring the correct placement of this family and, particularly, of the genus *Notocelis*. We have also used this dataset to infer for the first time diversification times within Acoelomorpha, which coincide with known bilaterian diversification and extinction events. Given the importance of morphological data in acoelomorph phylogenetics, we tested several partitions and models. Although morphological data failed to recover a robust phylogeny, phylogenetic placement has proven to be a suitable alternative when a reference phylogeny is available.

Xenacoelomorpha are mostly microscopic, benthic, marine worms, although some species can reach a larger size and two occur in freshwater habitats. Xenacoelomorphs are characterized by a simple morphology without many of the structures typical of other bilaterians, such as body cavity, through-gut, or circulatory and excretory systems (Jondelius et al. 2019). Within Xenacoelomorpha, the taxon *Xenoturbella* is the sister group of Acoelomorpha, which, in turn, consists of the two clades Acoela and Nemertodermatida. Morphological synapomorphies for Acoelomorpha were proposed by Ehlers (1985), and putative apomorphies between *Xenoturbella* and Acoelomorpha were discovered by Franzén and Afzelius (1987). Although the monophyly of Acoelomorpha and Xenacoelomorpha have been disputed in different phylogenetic studies, they have been strongly supported by phylogenomic studies and are widely accepted today (e.g., Philippe et al. 2011, 2019; Cannon et al. 2016; Laumer et al. 2019).

A cladistic analysis of morphological data led to the proposal of Acoelomorpha as an early bilaterian branch (Haszprunar 1996), which was later corroborated by molecular phylogenetics (Ruiz-Trillo et al. 1999; Jondelius et al. 2002). Subsequently, a detailed analysis of ultrastructural evidence caused Ehlers and Sopott-Ehlers (1997) to conclude that *Xenoturbella* is the sister taxon of all other Bilateria. However, there are two conflicting hypotheses regarding the position of Xenacoelomorpha within Metazoa, either sister group of other extant Bilateria (the Nephrozoa hypothesis) or nested within deuterostomes as sister to Ambulacraria (Xenambulacraria hypothesis). Both hypotheses have received support from phylogenomics (Hejnol et al. 2009; Philippe et al. 2011; Cannon et al. 2016; Laumer et al. 2019; Kapli et al. 2021; Mulhair et al. 2022) and analyses of gene content (Cook et al. 2004; Jiménez-Guri et al. 2006; Sempere et al. 2007; De Mendoza and Ruiz-Trillo 2011; Thomson et al. 2014; Juravel et al. 2023). Due to the implications for our understanding of animal evolution, this question has drawn a lot of attention, but phylogenetic relationships and evolutionary dynamics within Xenacoelomorpha remain relatively unexplored.

Within Xenacoelomorpha, the relationships among all six *Xenoturbella* species have been recovered with strong nodal support using complete mitogenome alignments (Nakano et al. 2017), but the more speciose Acoelomorpha (more than 400 species currently recognized; WoRMS Editorial Board 2024) poses a bigger challenge. Morphological descriptions are the basis of current familial classification (e.g., Westblad 1940; Dörjes 1968), and still represent an important tool for understanding phylogenetic relationships among acoelomorphs (Raikova et al. 1997; Hooge 2001). After the first molecular phylogeny was published (Hooge et al. 2002), an integrative approach combining molecular and morphological data was often employed (Petrov et al. 2004; Raikova et al. 2004; Tekle et al. 2005). This allowed the identification of a set of phylogenetically informative morphological characters such as body wall musculature and sperm ultrastructure. However, taxon sampling was limited in these studies since morphological studies of these microscopic soft-bodied animals are time-consuming, and generating molecular data required the collection of new specimens. In a study by Jondelius et al. (2011), three genes (18S, 28S, and COI) from about one-third of the clade’s diversity were used to reconstruct the phylogeny of Acoela. Similarly, Meyer-Wachsmuth and Jondelius (2016) also used three genes (18S, 28S, and Histone 3) to resolve the relationships within Nemertodermatida, including all but two of the accepted species at the time. These are the hitherto most comprehensive studies of acoel and nemertodermatid relationships. Subsequent studies have been mostly focused on revising intrafamilial relationships and describing new species (Kånneby et al. 2015; Atherton and Jondelius 2022).

Despite the phylogenetic framework provided by these studies, some suprafamilial relationships remain poorly supported, for example, the relationship of Dakuidae with the clade (Convolutidae + Mecynostomidae) in Acoela, or the relative position of *Nemertodermatida* and *Meara* in Nemertodermatidae. Likewise, some of the nodes contradict previous hypotheses based on morphological observations. For instance, Todt (2009) proposed pharynges in those acoels that possess them are independently evolved, whereas Jondelius et al. (2011) inferred the pharynx as an ancestral feature of acoels. Testing and resolving these relationships requires phylogenetic hypotheses supported by more data. Yet, despite the steady generation of genomic data from Acoelomorpha (Brauchle et al. 2018; Duruz et al. 2020; Abalde et al. 2023) and the advent of sophisticated phylogenomic methods for resolving contentious relationships at deep time scales (e.g., Mongiardino Koch and Thompson 2021; Irisarri et al. 2022; Song et al. 2023), this approach remains unexplored in Acoelomorpha.

Here, we have leveraged all available transcriptomes from this group and complemented them with newly generated ones (effectively doubling the number of sequenced species) to infer the first acoelomorph phylogeny based on genomic data. In doing so, we not only generated a robust phylogeny but also explored the data to identify potential sources of incongruence that generate topological instability. We then used this new phylogenomic hypothesis to 1) date major cladogenetic events within Acoelomorpha, 2) evaluate the performance of available morphological characters when reconstructing acoelomorph phylogeny and in phylogenetic placement of new species, and 3) study morphological evolution in this group.

## Material and Methods

### Data Generation

A total of 29 transcriptomes were generated in three separate batches from individuals collected between 2007 and 2020, preserved in either RNAlater or RNA Shield and long-term stored at -20 °C. Total RNA was extracted using the Zymo Microprep Quick-RNA kit (Zymo Research) and amplified with the SMARTer Universal Low Input RNA Kit (Takara Bio). The quality of the resulting cDNA was ensured with the Bioanalyzer High Sensitivity DNA Analysis and sent to either SciLifeLab or Macrogen for sequencing on an Illumina HiSeq X platform. The final dataset, including 19 transcriptomes downloaded from GenBank, consisted of 48 transcriptomes from 41 species: 33 Acoela, seven Nemertodermatida, and *Xenoturbella profunda* as outgroup (Supplementary Table S1).

Three cleaning and assembly strategies were devised to maximize assembly completeness. All transcriptomes were assembled following three pipelines and the best assembly was selected based on its completeness score, measured with BUSCO (version 3.0.2), and the Metazoa_odb9 database (Seppey et al. 2019). First, following standard practice, raw reads were cleaned with Trimmomatic and assembled with Trinity (version 2.9.1; Grabherr et al. 2011) with default parameters. Second, transcriptomes were assembled using the TransPi pipeline (version 1.1.0; Rivera-Vicéns et al. 2022) with three kmer lengths (21, 31, and 41). Third, raw reads were quality-filtered before the Trinity assembly in a three-step process: sequencing errors were corrected with Rcorrector (version 1.0.4; Song and Florea 2015), sequencing adapters removed with Trimmomatic (as implemented in Trinity (version 2.9.1)), and the reads were quality filtered with Prinseq (version 0.20.4; Schmieder and Edwards 2011), trimming nucleotides under 30 PHRED from both ends and filtering out reads with a mean quality under 20, entropy under 50, and shorter than 40 base pairs. Redundant contigs were removed with EvidentialGene (v2019.05.14; Gilbert 2016). After selecting the best assembly per transcriptome, cross-contaminants were filtered with CroCo (version 1.1; Simion et al. 2018) measuring the contig expression with Kallisto (version 0.46.2; Bray et al. 2016). Finally, coding regions with a minimum length of 300 amino acids were extracted with TransDecoder (version 5.3.0; Haas and Papanicolau 2010) and duplicates collapsed (minimum identity 95%, minimum overlap 40 amino acids) with the Dedupe program from BBMap (version 38.92; Bushnell 2014). The 300 amino acid TransDecoder threshold, which was implemented after initial experimentation with cutoffs ranging between 100 and 700 aa, was found to yield a useful number of orthogroups amenable to downstream analyses.

### Molecular Phylogenomic Analyses

The extracted proteins were assigned to orthogroups with OrthoFinder (version 2.4.1; Emms and Kelly 2019) and screened for paralogs with PhyloPyPruner (version 1.2.3; Thalén et al. 2021) with the following settings: pruning algorithm “Largest Subtree,” keep orthogroups with at least five taxa, trim branches longer than five times the standard deviation of all branch lengths, collapse nodes with nodal support under 60, and, in species-specific duplications, keep the sequences with the shortest pairwise distance to its sister taxa. For the seven species represented by two transcriptomes, only the specimen with the highest number of orthologs was kept. Non-homologous stretches within the sequences were identified and masked with Prequal (version 1.02; Whelan et al. 2018), and all sequences shorter than 250 unmasked amino acids were removed. All remaining orthogroups with more than five species were aligned with MAFFT (version 7.475; Katoh et al. 2009) using the L-INS-i algorithm. Ambiguously aligned positions, sequences shorter than 66% of the total alignment length, and sites with more than 80% missing data were filtered with BMGE (version 1.12; Criscuolo and Gribaldo 2010). Exploratory analyses revealed two sets of orthogroups within the species *Faerlea glomerata*, recovered deeply nested within the family Proporidae (this species was previously assigned to Isodiametridae and subsequently Mecynostomidae), suggesting potential contamination of this sample. Clan_check, designed to remove deep paralogy based on known monophylies in the tree (Siu-Ting et al. 2019), was used to screen all gene trees and flag all genes where *Faerlea* was recovered within Proporidae. The *Faerlea* sequences were removed and the genes were re-aligned and filtered with BMGE. Finally, the alignments that did not meet the assumptions of stationarity and homogeneity were identified with IQ-TREE (version 2.1.3; Minh et al. 2020) and removed. The resulting dataset included 2774 orthogroups.

The species *Notocelis gullmarensis* is known to be problematic due to long-branch attraction artifacts (LBA) that can hamper phylogenetic inference (Jondelius et al. 2011). Hence, an alternative version of this dataset was created after removing this species and realigning the relevant genes to infer the phylogenetic relationships within Acoelomorpha. Only 2764 alignments had at least five species after removing *Notocelis*. This dataset was used to infer a backbone phylogenetic tree. The original dataset including *Notocelis* was used afterwards to infer the position of this species and date the main cladogenetic events (see following section).

In order to alleviate the misleading effect of systematic bias in the data, the R script *genesortR* (Mongiardino Koch 2021) was used to measure several gene properties, aiming to subsample a subset of genes that minimize the presence of systematic bias while maximizing the phylogenetic signal. However, the genes that could minimize the presence of systematic biases were also the least informative ones, and genesortR failed to accommodate both parameters (minimize biases, maximize signal). Instead, we decided to try other common approaches to data filtering and created independent matrices after filtering by occupancy, substitution rate, level of saturation, compositional heterogeneity, and average patristic distances (as an indicator of LBA). Two matrices per filter were created, keeping the best 567 and 300 orthogroups from each of them. Unfortunately, the highly incomplete transcriptome from *Sterreria* sp. (SRR2682099), present in only nine alignments, was lost after filtering, but the presence of three other species ensured inferring genus-level phylogenetic relationships within Nemertodermatidae. In all datasets, orthogroups were concatenated into a supermatrix with FASconCAT-G (version 1.0; Kück and Longo 2014) and analyzed using coalescence and partition analyses with ASTRAL-III (version 5.7.7) and IQ-TREE (version 1.6.12), respectively (Nguyen et al. 2015; Zhang et al. 2017). Gene trees for the ASTRAL analysis were inferred with IQ-TREE (version 1.6.12), using ModelFinder to infer the best-fit model for each alignment. In an attempt to capture the complexity of the data, two site-specific models with 20 (C20) and 60 (C60) amino acid profiles were applied in IQ-TREE (Quang et al. 2008), using as a guide a phylogenetic tree inferred under a simple LG model with empirical amino acid frequencies and discrete gamma (LG + F + G) but no partitions. Finally, the matrix formed by the 300 alignments with the highest occupancy was analyzed with PhyloBayes running four independent chains under the CAT-GTR model, with four gamma categories, and keeping the invariable sites until reaching convergence (as per the built-in *bpcomp* and *tracecomp* commands).

We attempted to replicate all these analyses with the genes coded as nucleotides instead of amino acids, but the dataset was almost completely saturated and the inferred topologies failed to recover well-established monophylies such as that of the clade Nemertodermatida. Hence, these analyses were not considered any further.

### Placing Notocelis and Identifying the Sources of Incongruence

The original orthogroups including *N. gullmarensis* were analyzed to investigate their position in the Acoelomorpha phylogeny. The first step was to infer the phylogenetic position of *N. gullmarensis* with ASTRAL-III, the partition, C20, and C60 models of IQ-TREE, and PhyloBayes. The approximate unbiased (AU) test implemented in IQ-TREE was used to test the support of *Notocelis* branching from nine internal branches of the tree and to generate the site- and gene-wise support of each topology, reducing the alternative positions of *Notocelis* to four possibilities, as sister to Convolutidae, Mecynostomidae, both, or Dakuidae. Finally, the likelihood-mapping algorithm implemented in IQ-TREE (Nguyen et al. 2015) was used to create a new matrix, formed only by the alignments with over 70% of the quartets supporting one of these topologies. Then, the same phylogenetic analyses and topology tests were applied. A new topology test algorithm called MAST was released in IQ-TREE 2 as we were exploring this question (Wong et al. 2024). Unlike traditional AU tests, MAST can accommodate complex evolutionary models, in theory resulting in more accurate measures of support. Thus, the same four topologies were tested under the six models implemented: 1) unlink all parameters, 2) link rate heterogeneity across sites (gamma model), 3) link amino acid frequencies, 4) unlink only the substitution model, 5) unlink only the gamma model, and 6) link all parameters. Due to the presence of missing data in the matrix, branch lengths were linked across topologies (R. Lanfear, personal communication).

Unfortunately, despite the general support of the phylogenetic analyses of *Notocelis* being sister to the clade Convolutidae + Mecynostomidae, the topological support is not conclusive. Thus, we decided to investigate if introgression or hemiplasy could be the source of the observed incongruence. Two approaches of the *D* test were employed to accommodate the two recovered topologies. First, *D*_FOIL_, specifically designed to test introgression in a five-taxon, symmetric topology (Pease and Hahn 2015), was used with the IQ-TREE topology ((Convolutidae, Mecynostomidae), (Dakuidae, “Nadinidae”), Outgroup). A traditional *D*-statistic analysis was performed with the four-taxon topology (((Convolutidae, Mecynostomidae), Dakuidae), Outgroup) recovered by ASTRAL and PhyloBayes. One supermatrix for each three-species combination (one per family, 75 matrices) plus *Aphanostoma pulchra*, the selected outgroup for its high completeness, was created by filtering out the genes where not all four species are present and removing all other species. All matrices were analyzed with the R package *evobiR* (Jonika et al. 2023). As a control, the same process was repeated to test the presence of introgression from increasingly distant species (125 matrices): *Eumecynostomum macrobursalium* and *Aphanostoma virescens* (“Nadinidae,” same outgroup), *A. pulchra*, and *Haploposthia rubropunctata* (Isodiametridae, outgroup: *Diopisthoporus gymnopharyngeus*), and *D. gymnopharyngeus* (Diopisthoporidae, outgroup: *Meara stichopi*). Finally, the presence of hemiplasy was tested by measuring the Hemiplasy Risk Factor (HRF, “the fraction of incongruence expected to be due to hemiplasy”) using the R package *pepo* for all non-terminal branches of the ASTRAL-III tree inferred from the dataset including the 567 most complete orthogroups (Guerrero and Hahn 2018). Several mutation rates were used to calculate the HRF, including some higher than the ones estimated by MCMCtree (0.0004–0.001): 0.0001, 0.0005, 0.001, 0.005, 0.01, 0.05, and 0.1.

### Divergence Time Estimation

Divergence times within Acoelomorpha were inferred in a Bayesian framework with the MCMCtree package implemented in PAML (version 4.10; Yang 2007) based on secondary calibrations due to the absence of fossils from this group. These calibrations were based on a bilaterian time tree inferred from 18 genomes from 10 phyla, which included two acoel and one nemertodermatid species (Supplementary Table S2). The annotated proteins were clustered in orthogroups with OrthoFinder (version 2.4.1; Emms and Kelly 2019). Single-copy orthologs present in at least two phyla and three species were aligned with the L-INS-i algorithm implemented in MAFFT (version 7.475) and filtered with BMGE (version 1.12), removing all ambiguously aligned positions, sequences shorter than 66% of the total alignment length, and sites with more than 80% missing data (Katoh et al. 2009; Criscuolo and Gribaldo 2010). Phylogenetic inference was performed with a subset of 50 randomly selected orthogroups analyzed as independent partitions in IQ-TREE (version 1.6.12; Nguyen et al. 2015) and inferring the best-fit substitution model with ModelFinder. Branch lengths were used to estimate the prior for the mean substitution rate (rgene_gamma = 2 825 1). To infer the divergence times, we assumed an uncorrelated lognormal clock and a uniform distribution of the birth-death rates (*λ* = *μ* = 1, and *ρ* = 0). A WAG model with five gamma categories was applied to all partitions. Five fossil calibrations were used (Supplementary Table S3), following a uniform distribution and allowing a 5% chance of the inferred age to fall outside this range. The maximum age of the root was set to 650 Ma. Due to the uncertain position of Xenacoelomorpha in the metazoan tree, either as a sister to Nephrozoa or Ambulacraria, we inferred the divergence times over the two trees. We ran two independent chains for 11 million generations, sampling every 100 generations and discarding the first million as burn-in. Tracer (Rambaut et al. 2018) and the R package MCMCtreeR (Puttick 2019) were used to ensure chain convergence and that all ESS parameters were above 200.

Based on these results (Supplementary Fig. S1), the acoelomorph phylogeny inferred with IQ-TREE and including *N. gullmarensis* as sister to Dakuidae was calibrated in three nodes: the root (between 540 and 635 Ma), and the splits Acoela—Nemertodermatida (500–575) and *Praesagittifera naikaiensis*—*Symsagittifera roscoffensis* (175–200). These ages were defined based on the upper and lower limits of the 95% credible interval from the Xenambulacraria and Nephrozoa chronograms and followed a uniform distribution, allowing a 15% chance of falling outside this range. Only the 50 most complete orthogroups, analyzed as independent partitions, were used. The same MCMCtree parameters as above were used, but changing the prior from the mean substitution rate (rgene_gamma = 2 1524 1).

### Phylogenetic Inference with Morphological Data

A morphological matrix including all species and up to 44 characters was prepared based on descriptions from the literature and photographs of the specimens analyzed here (Supplementary Table S4). Several partition schemes were tested to maximize the phylogenetic signal of the data (Supplementary Table S5), such as 1) all characters together; 2) 10 random partitions, expected to work as a null model; 3) two partitions grouping characters from the same anatomical structure, but with two levels of detail; 4) assigned by PartitionFinder based on Akaike Information Criterion (AIC) or Bayesian Information Criterion (BIC) and linking or unlinking branch lengths (this resulted in two schemes); 5) by homoplasy scores (i.e., a proxy for the evolutionary rate of each character); and 6) all characters separated. Incidentally, homoplasy scores were measured as TNT “Inferred Weights” (Goloboff 1993) with a concavity parameter of three. The weight of each character was measured over a set of parsimony trees inferred from sectorial searches and tree fusing over 20 rounds. Four partitions were defined as the best scheme after comparing the total sum squared of all options between one and 10 clusters, and characters were assigned to each partition based on *k*-means.

The stepping-stone algorithm implemented in MrBayes (version 3.2.7; Ronquist and Huelsenbeck 2003) was used to calculate the likelihood of each scheme under the standard discrete model, but applying the ascertainment bias correction and with different model parameters: fixed or variable rates among partitions (among-partition rate variation: APRV), fixed or variable rates among characters (among-character rate variation: ACRV), and linking or unlinking branch lengths, testing nine models per partition scheme. Finally, the best overall partition scheme and best model configuration per scheme were identified with BayesFactors. MrBayes was used to infer a phylogenetic tree for each partition scheme, applying the best-fit model configuration. For each analysis we ran two independent runs with four Markov chains each for 50 million generations, sampling every 10,000 generations and discarding the first 25% as burn-in. Chain convergence was assessed by ensuring a correct mixing in the log-likelihood plot, that all ESS values were above 200, and that the Potential Scale Reduction Factor was at least one.

In addition, the ability of these characters to place a set of species in a given tree was tested using the phylogenetic placement algorithm implemented in RAxML (8.2.12; Stamatakis 2014). First, morphological characters were weighted in RAxML using the IQ-TREE topology as a guide tree with four gamma categories and applying the Lewis ascertainment bias correction. Then, a morphological matrix with 84 acoel species was downloaded from Jondelius et al. (2011) and used to place the species in the reference tree applying the inferred character weights.

### Morphological Evolution

The morphology of the ancestral acoelomorph was inferred from the same morphological matrix described above and using three methods. The IQ-TREE topology with *N. gullmarensis* as a sister to Dakuidae was selected as our working hypothesis. First, ancestral character states were inferred in Mesquite (version 2.75; Maddison and Maddison 2018) using the maximum likelihood method and the stored probability models, only considering characters with a likelihood over 0.69. The second method was the function *estimate_ancestral_states* implemented in the R package *Claddis* (version 3.6.1; Lloyd 2016) with default parameters, which include treating all characters with polymorphisms or missing as having equal probabilities. Finally, we also used BayesTraits (version 4.0; Pagel and Meade 2006) applying the multistate model. We ran two independent chains for 50 million generations, sampling every 10,000 generations and discarding the first 10% as burn-in.

The function *test_rates* implemented in *Claddis* was used to calculate the evolutionary rate of all species and characters and identify potential rate shifts. The morphological matrix was edited to change the missing data by uncertainties (all states have equal probability) and leaving inapplicable characters as missing. Potential rate shifts along the tree were calculated based on five different partitions: 1) all branches separated, 2) all species separated, 3) all families separated, and separating acoels from nemertodermatids including 4) or not 5) the branch connecting them to the root. The likelihoods of all partitions were compared using the weighted Akaike Information Criterion as implemented in *Phytools* (version 1.5-1; Revell 2012). The same approach was used to infer shifts in the evolutionary rate of the characters, in this case, divided into two partition schemes, such as 1) all characters separated, and 2) grouping characters from the same structure (“Morphological partition 2” in Supplementary Table S5).

Finally, we visually compared the pairwise morphological and molecular distances among species. Untransformed morphological distances were calculated with the function *calculate_morphological_distances* implemented in *Claddis* using the Maximum Observable Rescaled Distance (Lloyd 2016), which modified the Gower Coefficient (Gower 1971) to be on a zero to one scale. Inapplicable states were considered during distance calculation. Molecular distances were calculated from the 50 most complete genes with the function *dist.ml* implemented in *Phangorn* (version 2.11.1; Schliep 2011) and applying the LG model. In both cases, the distance of each species was summarized as the average pairwise distances from all other species in the tree. A scatterplot was created with *ggplot2* (version 3.4.2; Wickham 2016).

## Results

### Molecular Phylogenomics

The full dataset, after removing duplicate individuals and the species *N. gullmarensis*, was composed of 2764 OGs and 40 species. The species with the fewest orthogroups were the three *Sterreria* spp., all of them present in fewer than 20, while *Diopisthoporus* sp.1, *Flagellophora* sp., and the two *Solenofilomorpha* spp. were present in fewer than 100 alignments (Supplementary Table S1). Yet, all but two families (Ascopariidae and Solenofilomorphidae) were present in at least 442 orthogroups and roughly 75% of the genes were formed by at least three families.

**Figure 1.**
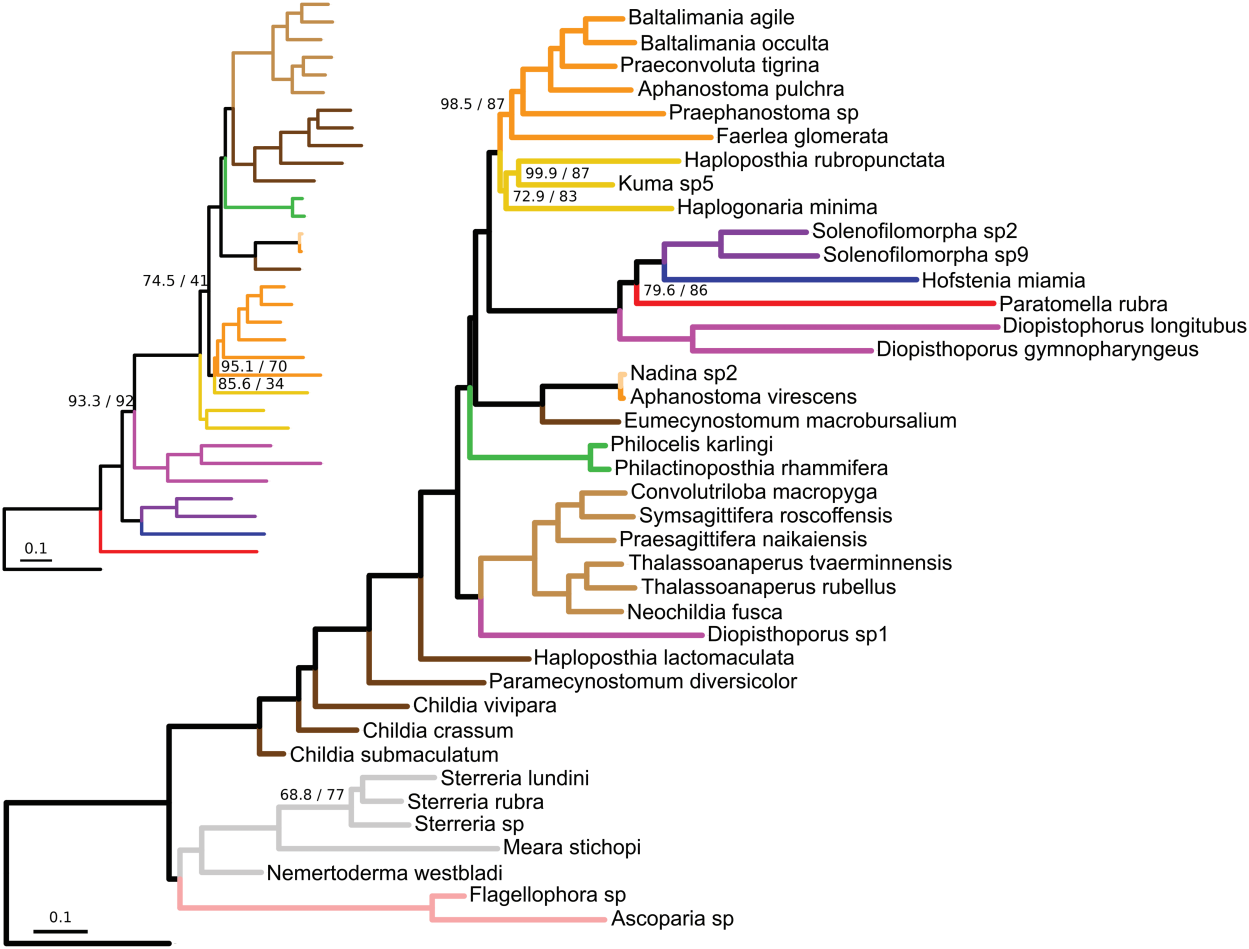
Phylogenomic tree showcasing the difficulty of inferring a robust tree from genes filtered by substitution rate. The tree was inferred from the 567 genes with lowest substitution rate, using site-specific models with 20 amino acid categories (C20) in IQ-TREE and using *Xenoturbella profunda* as the outgroup. Unless otherwise specified, all nodes have good support (ultrafast bootstrap/SH-like approximate likelihood ratio test > 95). The scale bar indicates substitutions per site. The inset to the left shows a tree inferred from the same matrix but after removing all Nemertodermatida species and using a site-homogeneous partition model.

**Figure 2.**
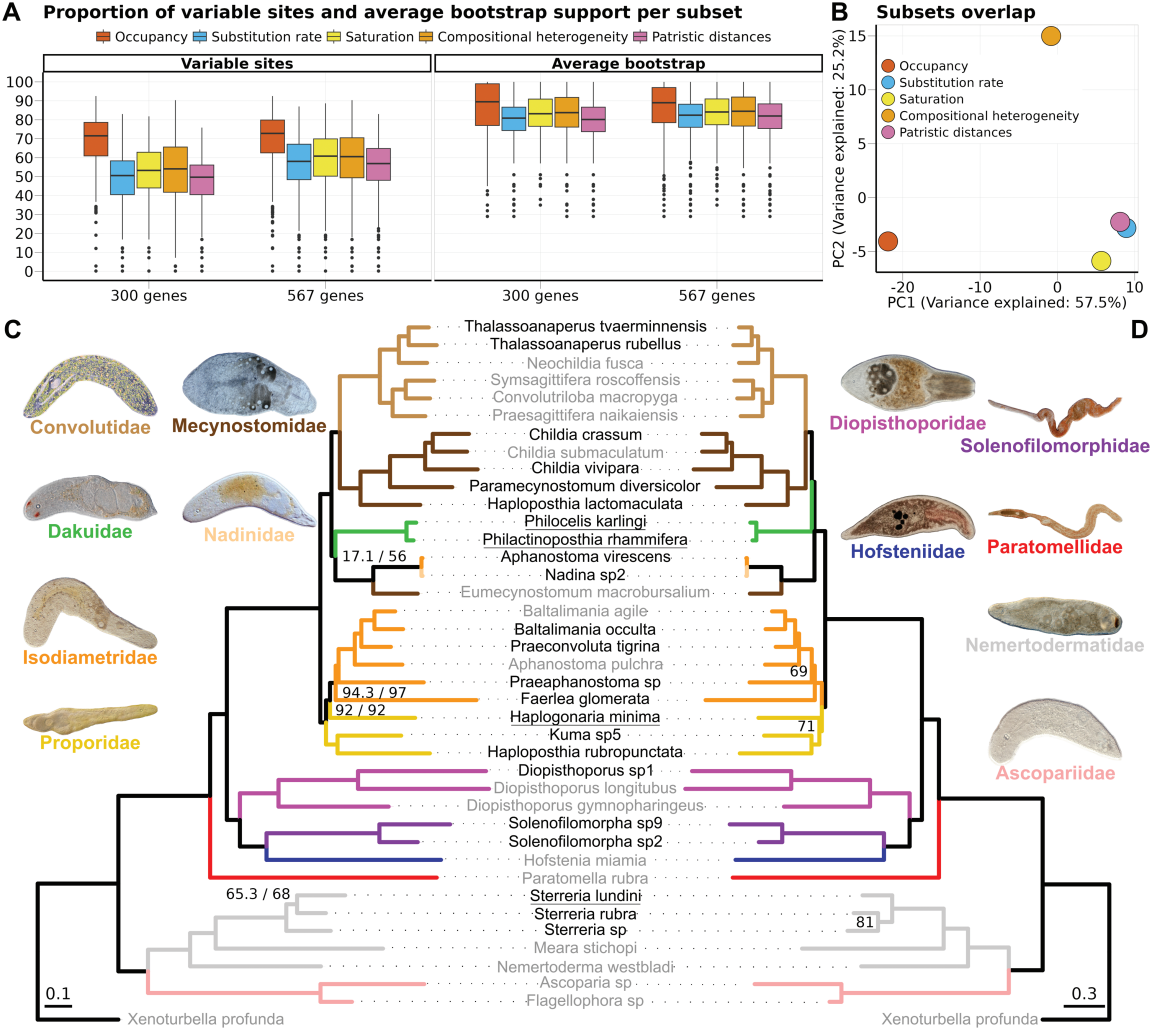
Summary of the phylogenomic analyses. a) Proportion of variable sites and average bootstrap support in the supermatrices filtered by occupancy, substitution rate, saturation, compositional heterogeneity, and patristic distances. b) Gene overlap among the same matrices, displayed as a PCA. The presence and absence of genes on each matrix were used to calculate the two main principal components. Phylogenomic trees inferred from the 300 most complete genes using IQ-TREE and 60 amino acid categories c) or PhyloBayes d). Unless otherwise specified, all nodes have maximum support (c: ultrafast bootstrap/SH-like approximate likelihood ratio test; d: posterior probabilities). Species names in gray indicate samples downloaded from the SRA, in black new transcriptomes, and names underlined highlight conflicts between the two topologies. The scale bars indicate substitutions per site.

This big dataset allowed us to test several filtering criteria: occupancy, substitution rate, saturation, compositional heterogeneity, and average patristic distances. All but occupancy rendered similar trees regardless of the algorithm and model (Fig. 1, Supplementary Figs. S2–4). However, the low support of monophyletic Nemertodermatida, paraphyletic Mecynostomidae, and the general disagreement with previous phylogenies, made us discard phylogenies inferred with all filter types except occupancy as artefactual. These trees were similar to the phylogenies inferred from nucleotide data, which presented high saturation rates (over 75% in 90% of the orthogroups). The matrices filtered by rate, saturation, patristic distances (which are broadly overlapping, Fig. 2b), and compositional heterogeneity have generally fewer variable sites and lower bootstrap support than the occupancy matrices (Fig. 2a), which recovered strongly supported trees. Reanalysis of the problematic matrices after removing all nemertodermatid species recovered topologies closer to the occupancy datasets, revealing a long branch attraction artifact, but some topological differences remain (Fig. 1, Supplementary Figs. S2–4).

The two occupancy datasets returned robust phylogenies in all analyses but with slight topological differences depending on the algorithm (Fig. 2c and d), and generally recovered the monophyly of Nemertodermatida, Acoela, and all families analyzed. Within Acoela, the first off-shoot is *Paratomella*, followed by the pharynx-bearing families with Diopisthoporidae as sister to the Hofsteniidae + Solenofilomorphidae clade. Next, maximum likelihood recovered three pairs of families, in order: Proporidae + Isodiametridae, Dakuidae + Nadinidae, and Mecynostomidae + Convolutidae (Fig. 2c), whereas ASTRAL and PhyloBayes (the latter based only on the 300 genes matrix) recovered Dakuidae as sister to the Mecynostomidae + Convolutidae clade (Fig. 2d). The other two differences observed are the relationships among *Sterreria* species and the position of *Haplogonaria minima* (Proporidae), recovered as sister to Isodiametridae and not the other proporids by IQ-TREE. Nevertheless, all three differences correspond to nodes with low statistical support in at least one of the analyses. The strongly supported clade, herein called “Nadinidae,” consists of the species *Nadina* sp. (Nadinidae), *Aphanostoma virescens* (Isodiametridae), and *Eumecynostomum macrobursalium* (Mecynostomidae). We used the name Nadinidae due to the inclusion of a specimen identified as *Nadina* sp. based on similarities with the type species of that genus, but the original description (Uljanin 1870) is lacking in detail, and analysis of fresh material from the type locality would be desirable to determine the taxonomic status of this family. *Aphanostoma virescens* has a highly characteristic pigment pattern and is easily identified. *Eumecynostomum macrobursalium* is one of several species classified within Mecynostomidae that are in need of taxonomic revision. The maximum likelihood topology (Fig. 2c) was chosen as our working hypothesis in all downstream analyses due to its stronger support compared to the coalescence tree, which also inferred near-zero branch lengths in some nodes (Supplementary Fig. S5). Similarly, after 32,000 generations (and over 650,000 core-hours) PhyloBayes converged in the tree space (*bpcomp* maxdiff = 0.09) but failed to reach full convergence of the continuous parameters of the model.

Having inferred a robust phylogeny for Acoelomorpha, we focused on the position of *N. gullmarensis* (Supplementary Table S6). All phylogenetic analyses recovered *Notocelis* sister to the clade Convolutidae + Mecynostomidae. The only exception occurs when analyzing the occupancy matrix including the 567 most complete orthogroups, when it is recovered as sister to the other Dakuidae species. In turn, AU tests and site- and gene-wise analyses supported four to six alternative topologies, depending on the matrix. The newer MAST algorithm predominantly supports a sister relationship to Dakuidae or Nadinidae, and only one model (all parameters but amino acid frequencies unlinked) supported a closer relationship to Mecynostomidae.

**Figure 3.**
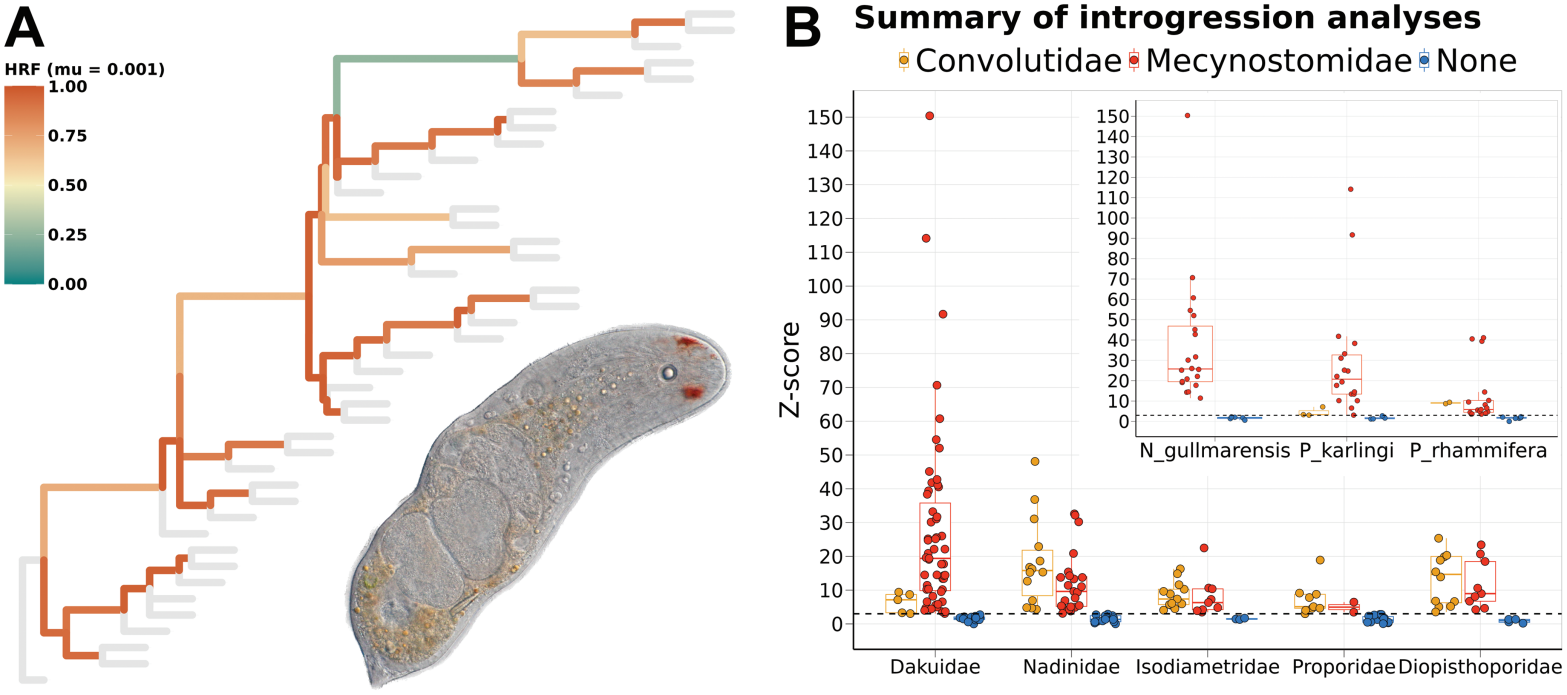
a) Hemiplasy Risk Factor calculated for all internal branches of the tree, based on the ASTRAL tree inferred from the 567 most complete genes and a 0.001 mutation rate. In the inset, a photo of the species *N. gullmarensis*. b) Comparison of the *z*-score calculated from all the analyzed submatrices separated per family. The inset to the right shows the results for the three species of the family Dakuidae. The dashed lines mark the significance threshold in the *z*-score (*z*-score = 3).

In an attempt to identify the source of this incongruence, we aimed to find a signal of introgression or hemiplasy in our data (Fig. 3). First, the Hemiplasy Risk Factor (HRF), calculated over the coalescence tree inferred from the 567 most complete genes, detected high rates (over 50%) of hemiplasy in all internal branches but the one leading to Convolutidae (Fig. 3a). The high HRF might be the reason behind the general difficulty in inferring a robust phylogeny as the data is filtered, but the HRF in the *Notocelis* branch is not higher than in other, fully resolved, parts of the tree. The *D*-statistic revealed a strong signal of introgression between Dakuidae and Mecynostomidae (Fig. 3b). As a control, we ran the same analysis over the increasingly distant families Nadinidae, Isodiametridae, Proporidae, and Diopisthoporidae. We did find some signal of introgression in all families, with *z*-scores generally higher than three, but they were not nearly as strong (i.e., the ABBA–BABA ratio deviates more from zero when Dakuidae is analyzed). Besides, all families but Dakuidae present similar *z*-scores among them and no preference for either Mecynostomidae or Convolutidae. If we consider the result of these families as a background signal, we still find some signal of introgression between Dakuidae and Mecynostomidae, which would explain the instability of these nodes among algorithms and the difficulty of placing *Notocelis* in the tree.

Finally, we checked if taxon-wise compositional heterogeneity (measured as the Relative Composition Frequency Variability, RCFV, in BaCoCa (v1.105.r; Kück and Struck, 2014)) could be another driver of topological discordance, but that does not seem to be the case. None of the acoels involved show a higher than average RCFV except for *Philactinoposthia rhammifera* (0.0027), but the other Dakuidae are among the species with the lowest RCFV (*Notocelis*: 0.0012, *Philocelis*: 0.0007). RCFV was generally higher in Nemertodermatida species, including *Sterreria*, but these are very incomplete transcriptomes (Supplementary Table S7).

### Divergence Time Estimations

**Figure 4.**
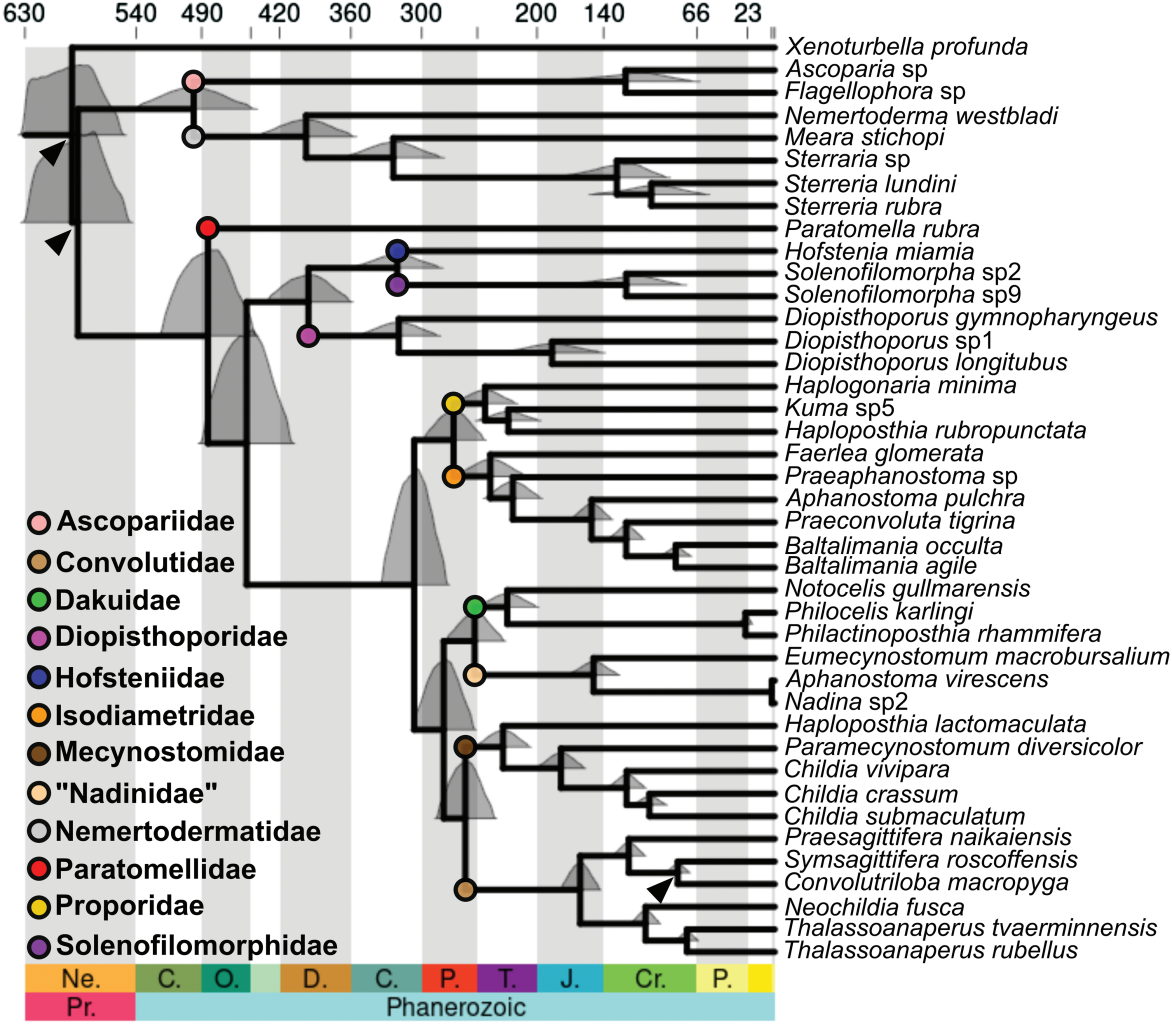
Chronogram inferred with MCMCtree, based on three secondary calibrations (highlighted with a black arrowhead) and the 50 most complete orthogroups. The origin of each family is marked with a circle, following the same color scheme from Figs. 1 and 2. The white and gray bars correspond to geological periods. The 95% credible interval of each node shows the full distribution of age estimates.

Major cladogenetic events within Acoelomorpha were inferred using an uncorrelated lognormal clock and a uniform distribution of the birth–death rates and based on three secondary calibrations (the root age and the splits Acoela—Nemertodermatida and *P. naikaiensis—S. roscoffensis*). MCMCtree reached full convergence, with an effective sampling size above 3000 in all parameters. The first cladogenetic events within Acoela (the branching of *Paratomella*) and Nemertodermatida (Ascopariidae—Nemertodermatidae) were estimated around the same time, roughly 490 million years ago (Ma; Fig. 4). Within Acoela, we see a clear boundary 250 Ma. The crown age of all families is older, whereas all splits among genera are younger. All species diversification events occurred in the last 200 Ma except *Diopisthoporus gymnopharyngeus* from the other 2 *Diopisthoporus* spp., estimated ca. 325 Ma. Within Nemertodermatida there is no such clear pattern. The origin of nemertodermatid genera was inferred between 400 and 300 Ma, whereas the *Ascoparia*—*Flagellophora* split was dated 130 Ma, roughly at the same time as the cladogenetic events within the genus *Sterreria* (Fig. 4). More generally, all cladogenetic events were dated over 75 Ma except for the separation between *Nadina* sp.—*A. virescens* and *Philocelis karlingi*—*P. rhammifera*.

### Morphological Phylogenetics

The best partition scheme was inferred by PartitionFinder, under a model with variable among partition rate variation, equal among character rate variation, and linked branch lengths (Supplementary Tables S8 and S9). Despite the statistically significant differences in model fit among partitions, all partitions recovered the same topology, with variations in branch length, largely incongruent with the molecular phylogeny (Supplementary Fig. S6). The only similarities between the two are the clade formed by the pharynx-bearing worms, with *Diopisthoporus* sister to *Solenophilomorpha* + *Hofstenia*, the relative proximity of the Convolutidae, Mecynostomidae, and Dakuidae species, and the proximity of all Isodiametridae species in the tree, although without forming a clade.

Despite the poor performance for confidently resolving phylogenetic relationships, morphological characters seem to be a good predictor of the phylogenetic position of a species when a backbone phylogeny is available (Table 1). After calibrating the weight of each character, the RAxML phylogenetic placement algorithm identified the correct position of 60 out of 83 species. Among the other 23, eight come from families not present in the tree. The worst family in terms of accuracy is Proporidae, with only five out of 13 species correctly placed. In Solefilomorphidae and Convolutidae, misplaced species are characterized by an important fraction of missing data.

**Table 1.** Summary statistics of the phylogenetic placement approach using morphological data. Bold rows highlight general stats and plain text family-specific data

Family	# Species	# Correct	Percentage
Actinoposthiidae^a^	5	0	0.00
Convolutidae	15	12	80.00
Dakuidae	7	6	85.71
Diopisthoporidae	1	1	100.00
Hallangiidae^a^	1	0	0.00
Hofsteniidae	1	1	100.00
Isodiametridae	19	18	94.74
Mecynostomidae	13	13	100.00
Otocelididae^a^	2	0	0.00
Paratomellidae	1	1	100.00
Proporidae	13	5	38.46
Solenofilomorphidae	5	3	60.00
**In the reference tree**	**75**	**60**	**80.00**
**Not in the reference tree**	**8**	**0**	**0.00**
**Total**	**83**	**60**	**72.29**

^a^Families not present in the reference tree.

### Ancestral Character State Reconstruction

Three alternative methods were used to estimate the ancestral morphology of Acoelomorpha, Acoela, and Nemertodermatida, based on the topology inferred by IQ-TREE with *Notocelis* placed as sister to Dakuidae. All three methods were affected by missing data, which was particularly obvious in the characters describing the wall muscles (Supplementary Table S10). The most complete reconstruction was performed by Mesquite, closely followed by *Claddis*, but BayesTraits returned a lot of uncertainty throughout the matrix despite the successful mixing of the chains. The ancestral acoelomorph was a relatively simple worm, cylindrically shaped, with frontal glands and a mouth in the ventral region, and separate male and female reproductive organs. This worm had paired testis, seminal vesicle, and male gonopore, as well as antrum and an unpaired ovary, without overlap between the testes and the ovaries. The wall musculature was similarly simple, with longitudinal muscles enwrapping the mouth, longitudinal muscles between the mouth and the frontal pore, and diagonal muscles in the dorsal side. The ancestral acoel and nemertodermatid were relatively similar, but the presence of antrum and vagina were unclear in the latter, the distribution of the wall muscles could not be inferred for any of the clades, and they differed in the presence of pigmentation (present in Acoela; Supplementary Table S10).

### Evolutionary Rates

The inferred chronogram and the morphological matrix were used to calculate the rate of morphological evolution of all species in the tree, as well as of each morphological character. *Claddis* detected an acceleration in the evolutionary rate of the sexual characters. The best-fit model compares them grouped in anatomical partitions, and the antrum is the only organ evolving at a faster rate (97% of the cumulative weight). When each character is analyzed independently, only the shape of the antrum and the glandulomuscular eversible copulatory organ evolve at accelerated rates (Supplementary Fig. S7a). Regarding the evolution of species, wAIC strongly supports a rate shift in the branch leading to Convolutidae, which is evolving at faster rates than other families (97% of the cumulative model weight; Supplementary Fig. S7b). Looking at the species-specific rates, 4 species might be outliers within their respective families, such as *Aphanostoma* (Isodiametridae), *Paramecynostomum* (Mecynostomidae), and *Meara* (Nemertodermatidae) show relatively high rates, whereas in *Praeaphanostoma* (Isodiametridae), it is comparatively slower (Supplementary Fig. S7b).

When we compared the average molecular and morphological distances among species (Supplementary Fig. S8), *Hofstenia* shows visibly higher morphological distances than the other pharynx-bearing worms, despite being closely related to *Solenofilomorpha*. All species from the family Isodiametridae are recovered tightly packed, both molecular and morphologically, except *Faerlea*, which shows higher morphological than molecular distance. Within Convolutidae, all species have almost identical average molecular distances, but are morphologically very different, their distances ranging from 0.26 (*Neochildia fusca*) to 0.45 (*Convolutriloba macropyga*).

## Discussion

One of the main challenges in building a backbone phylogeny for Acoelomorpha is gathering a taxonomically broad dataset. Here, we have leveraged years of sampling efforts to generate 29 new transcriptomes, more than doubling the number of available species (from 17 to 39) and increasing the number of sequenced families by 50%, up to roughly 70% of the family diversity. Despite the uneven completeness of the transcriptomes, we managed to generate a dataset with 2774 orthogroups, with more than 400 for all but two of the families. This is the largest dataset assembled to date to resolve phylogenetic relationships within Acoelomorpha.

### Incomplete Lineage Sorting and Introgression Might be the Main Drivers of Topological Instability

Genome-wide phylogenomics is often hampered by the presence of conflicting signal in the data, which may lead to erroneous or contrasting topologies (Telford et al. 2015). Common strategies to ameliorate the effect of systematic biases include filtering out alignments by properties, such as substitution rate, compositional heterogeneity, or saturation, individually or combined (e.g., Struck et al. 2014; Irisarri and Meyer 2016; Kocot et al. 2017; Cunha et al. 2022). Filtering by compositional heterogeneity, substitution rate, average patristic distance, or saturation led to less informative matrices (measured as the proportion of variable sites and average bootstrap support), in agreement with previous observations (Källersjö et al. 1999; Mongiardino Koch 2021), which resulted in generally unresolved trees. These matrices also suffered from long branch attraction, as revealed by removing the Nemertodermatida species from the matrix, which highlights the importance of outgroup selection (DeSalle et al, 2023). This problem was not observed in the occupancy-filtered datasets, which were the only ones capable of confidently resolving inter-family relationships when all taxa were considered. Filtering by occupancy also helps reduce the nonrandom distribution of missing data, which has been suggested to mislead phylogenetic inference (Streicher et al. 2016; Ballesteros et al. 2019; Uribe et al. 2022). Incidentally, it is interesting to note that filtering by rate, saturation, and average patristic distances resulted in matrices including almost the same genes, which stresses the importance of assessing the independence of the data when creating parallel datasets.

While the occupancy datasets successfully inferred strongly supported trees, it also returned two slightly different topologies (IQ-TREE vs. ASTRAL + PhyloBayes). One potential explanation would be the large amount of missing data, which is known to create “islands” of similarly scoring trees, hampering the exploration of the tree space (Sanderson et al. 2011; Roch and Steel 2015; Suvorov et al. 2022). This would support the summary topology inferred under the coalescence method as the preferred topology but does not explain the differences observed between site-specific models in maximum likelihood or Bayesian inference. Instead, we suspect incomplete lineage sorting (ILS) and introgression are major drivers of topological instability. Our analyses revealed a high risk of hemiplasy throughout the tree. The HRF was built under the assumption that either ILS or introgression are the only sources of incongruence, which differ in the length of the branches connecting discordant nodes (Guerrero and Hahn 2018). On one hand, we observe very short branches connecting the different families, which is particularly evident in the ASTRAL and PhyloBayes topologies, consistent with the presence of ILS (Singhal et al. 2021; Cunha et al. 2022), which causes low signal-to-noise ratios and can lead to contrasting topologies (Mongiardino Koch et al. 2023). On the other hand, we have also detected deep introgression events among families, with particular intensity between Dakuidae and Mecynostomidae. The position of Dakuidae and particularly *N. gullmarensis* are the two main differences between the two proposed topologies. Altogether, we hypothesize that ILS is the major driver behind the inconsistency between the species tree and the gene trees but that the main differences in the position of *Notocelis* are linked to introgression. However, in the only two phylogenetic studies that include sequences of *N. gullmarensis* (here and (Jondelius et al. 2011), this species is characterized by a long branch. An extended sampling of Dakuidae and in particular other *Notocelis* species could help stabilize this node (Bergsten 2005; Brinkmann et al. 2005; Uribe et al. 2019).

### Acoelomorpha Phylogenomics and the Reconstruction of the Ancestral Acoelomorph

Acoelomorph transcriptomes have hitherto been used in genome-wide phylogenomics to infer the position of this clade among bilaterians but with limited taxon sampling within the group (Cannon et al. 2016; Philippe et al. 2019). These analyses are generally congruent with our results, but they lacked several important taxa (e.g., none includes both *Paratomella* and *Diopisthoporus*). Inter-family relationships have been more carefully explored from PCR-amplified genes (Hooge et al. 2002; Jondelius et al. 2011; Meyer-Wachsmuth and Jondelius 2016). Among these, Jondelius et al. (2011), focused on Acoela, and Meyer-Wachsmuth and Jondelius (2016), on Nemertodermatida, both based on three genes, stand out as the most taxonomically sound. Our results tend to agree with theirs, with only minor differences among them. Within Nemertodermatida, Meyer-Wachsmuth and Jondelius (2016) also recovered the two families as monophyletic, but the relationships within Nemertodermatidae were unclear. In contrast, we always recover, with strong support, *Nemertoderma* as the first off-shoot within the family. Within the acoels, the observed differences are more important.

Currently, the most comprehensive phylogenetic study of Acoela includes 126 species, about three times more than here, from the same families as this except for Hallangiidae instead of Nadiniae (Jondelius et al. 2011). There are two main differences between the two studies. First, we recovered a clade formed by the families Isodiametridae and Proporidae that does not exist in Jondelius et al. (2011). Second, we did not recover *Diopisthoporus* as the sister taxon to all other acoels. Instead, *Paratomella* is here consistently recovered as the first off-shoot within Acoela, whereas *Diopisthoporus* forms a clade with the other pharynx-bearing acoels Solenofilomorphidae and Hofsteniidae. An early-branching *Paratomella* had already been suggested based on sperm morphology (Ehlers 1992), but *Diopisthoporus* was not included in that analysis. This difference has dramatic consequences in the reconstruction of the ancestral acoel, a core topic in Jondelius et al. (2011).

Based on our topology, the ancestral character state analyses recovered a simpler worm, with a ventral mouth, without pharynx, penis stylet, or vagina, and with ovaries posterior to the testes without any overlap, being the pharynx the most important difference. An ancestral pharynx was strongly supported by Jondelius et al. (2011) in conflict with hypotheses stating non-homology of acoel pharynges based on ultrastructural differences (Todt 2009). The pharynges of *Hofstenia* and *Solenofilomorpha* are similar, which led to a hypothesis of a close relationship between the two families. On the other hand, an independent origin of the pharynx in other species, such as *Proporus bermudensis*, is likely.

### Morphological Phylogenetics Do Not Confidently Resolve Acoelomorph Relationships

Molecular phylogenomics has become the main tool for inferring phylogenetic relationships among organisms. However, the intrinsic value of morphological evidence should not be overlooked (Lee and Palci 2015). In Xenacoelomorpha, many of the currently accepted species are only known from their type localities, and there are numerous monotypic genera and families. Incorporating all of these in a phylogenomic study would require a monumental collecting effort. In these and other cases, morphological descriptions become an important tool for inferring their closest relatives (e.g., Raikova et al. 2004; Hooge and Tyler 2005; Tekle et al. 2005). Thus, carefully evaluating the phylogenetic potential of morphological data is imperative.

As with molecular data, structuring morphological characters into partitions has been shown to strengthen phylogenetic inference (Clarke and Middleton 2008; Tarasov and Génier 2015; Rosa et al. 2019). Here, all partition schemes recovered the same partially unresolved topology, suggesting that the phylogenetic signal of the matrix is strong enough to overcome model misfit. The morphological tree also recovered the monophyly of the pharynx-bearing families in concordance with the phylogenomic analyses, albeit with low support.

In an attempt to maximize the usefulness of morphological data for inferring phylogenetic relatedness, we tested a phylogenetic placement approach. To counteract the misleading signal observed in the morphological data, we weighted all characters in the matrix to prioritize those that are more consistent with our phylogenetic hypothesis (Berger and Stamatakis 2010). This approach placed over 70% of the taxa within the correct family. One-third of the misplaced species are classified in families not represented in the reference phylogeny. Although we present the most complete acoelomorph tree inferred to date (previously, Jondelius et al. (2011) included more acoel species, but not families), we still lack 30% of the family diversity. Future sampling efforts should focus on underrepresented taxa to maximize family diversity. Seven out of the 15 misplaced species are characterized by an important fraction of missing data due to incomplete species descriptions and lack of detailed morphological studies using electron microscopy and immunocytochemistry. The other eight are classified in the family Proporidae, which is notoriously variable morphologically and in dire need of taxonomic revision.

### First Estimate of Acoelomorph Divergence Times

We dated the main cladogenetic events within Acoelomorpha using secondary calibrations generated from a bilaterian chronogram under both the Nephrozoa and Xenambulacraria hypotheses, which we inferred from available genomes. Three considerations need to be taken into account when interpreting the results. First, we have found a strong signal of topological incongruence between gene trees and the species tree throughout our phylogeny. Topological incongruence is known to cause inaccurate substitution rate estimations (Mendes and Hahn 2016), biasing estimates of divergence times (Carruthers et al. 2022). Here, this would translate into shorter internal and longer terminal branches. Second, this analysis is sensitive to rate variation among lineages and to the number and quality of the calibrations (Filipski et al. 2014; Soares and Schrago 2015; Zheng and Wiens 2015). Since our analysis is restricted to two calibration points close to the root and one very shallow, it is difficult to infer internal rate shifts. Third, secondary calibrations are known to produce less accurate inferences due to the cumulative uncertainty of two consecutive estimates (Hipsley and Müller 2014; Schenk 2016).

With its limitations, our analysis is the current best effort to date cladogenetic events in Acoelomorpha, which co-occur with known bilaterian diversification and extinction events. We observe two contrasting patterns between Nemertodermatida and Acoela, probably due to the larger diversity of the latter. In both cases, there is a period of roughly 100 million years between the split of the two clades and the origin of the first families, but Nemertodermatida is characterized by long periods of stasis not observed in Acoela. Although the early diversification of Acoela is relatively slow, there is an acceleration in the Permian, when we observe the origin of most of the families, that continues throughout the Triassic, when almost half of the genera originated. This timeframe coincides with important climatic events, including the Late Paleozoic Ice Age, the Carboniferous-Permian Biodiversification Event and the Late Early Triassic radiation (Fan et al. 2020; Shi et al. 2021). These quick radiations might explain the short internal branches connecting the different families, which could explain the observed gene tree discordance (Pardo-De la Hoz et al 2023). Moreover, there is a period of 50 Ma without any diversification events around the Triassic-Jurassic transition, which could potentially be attributed to the end of the Triassic mass extinction (Raup and Sepkoski 1982).

### Convolutidae Are Fast-Evolving Acoels

We also used this chronogram to infer the rates of morphological evolution in acoelomorphs and found Convolutidae to evolve at faster rates than other families. Convolutids have a number of innovations that may have accelerated diversification and morphological evolution (Jondelius and Jondelius 2019). They have symbiotic algae supplementing their otherwise carnivorous lifestyle and they have successfully diversified in the phytal. Some of them, for example, *Symsagittifera* and *Convolutriloba*, reproduce asexually, which is otherwise very rare in Acoelomorpha. Furthermore, in some Convolutidae there are unique structures such as sagittocysts, multiple bursal nozzles, and other accessory reproductive organs. Some of the species often occur in very high numbers in easily accessible litoral locations and some species such as *Symsagittifera roscoffensis* and *Convolutriloba* spp have become models for Acoelomorpha genomics and development (Shannon and Achatz 2007; Martinez et al. 2021). Two characters associated with the male reproductive organ, the shape of the antrum and the glandomuscular eversible copulatory organ, were estimated to evolve at faster rates. This is consistent with their utility to taxonomically distinguish species and also with sexual selection in connection with internal fertilization.

## Supplementary Material

Data available from the Dryad Digital Repository: https://dx.doi.org/10.5061/dryad.nvx0k6f0j.

syae057_suppl_Supplementary_Material_Figures_S1-S8

syae057_suppl_Supplementary_Material_Tables_S1-S9

## Data Availability

New transcriptomes have been deposited on GenBank (Bioproject PRJNA1106782). The code necessary to replicate these results has been uploaded to Zenodo (https://doi.org/10.5281/zenodo.13845748) and GitHub (https://github.com/saabalde/2024_Acoelomorpha_phylogenomics).
