# Supplementary material for: A Phylogenomic Backbone for Acoelomorpha Inferred From Transcriptomic Data
Source: Syst Biol. 2024 Oct 25;74(1):70–85. doi: 10.1093/sysbio/syae057 (PMC11809588; doi:10.1093/sysbio/syae057)
Supplement: syae057_suppl_Supplementary_Material_Figures_S1-S8 [file syae057_suppl_supplementary_material_figures_s1-s8.docx]

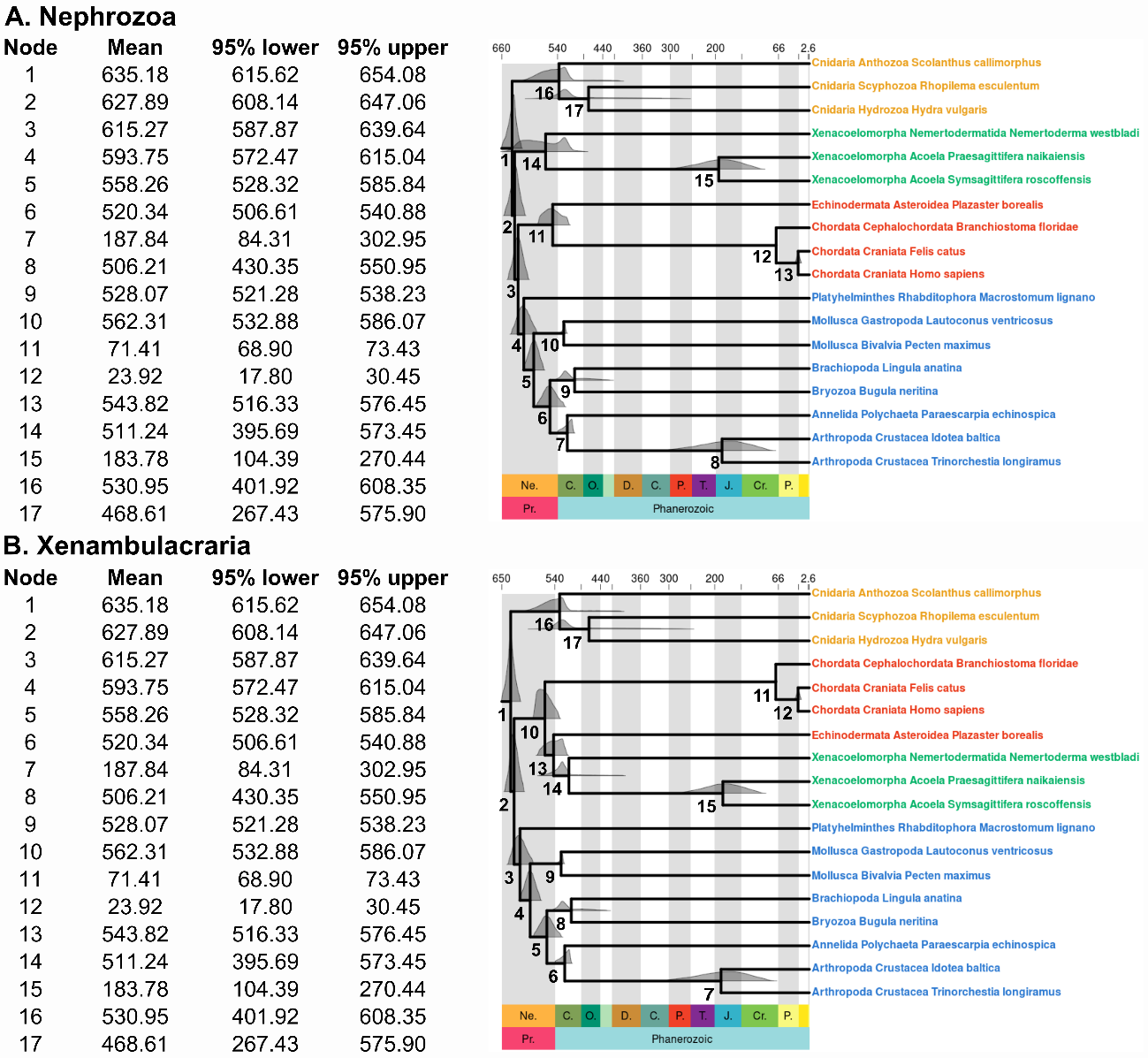
**Supplementary Figure S1** – Bilaterian chronogram, including the two debated positions of Xenacoelomorpha. Color tips indicate the main animal groups: Yellow - Cnidaria, Red - Chordata, Blue - Protostomia, and Green - Xenacoelomorpha. The table to the left indicates the mean divergence times and the upper and lower limits of the 95% credible interval of each node.


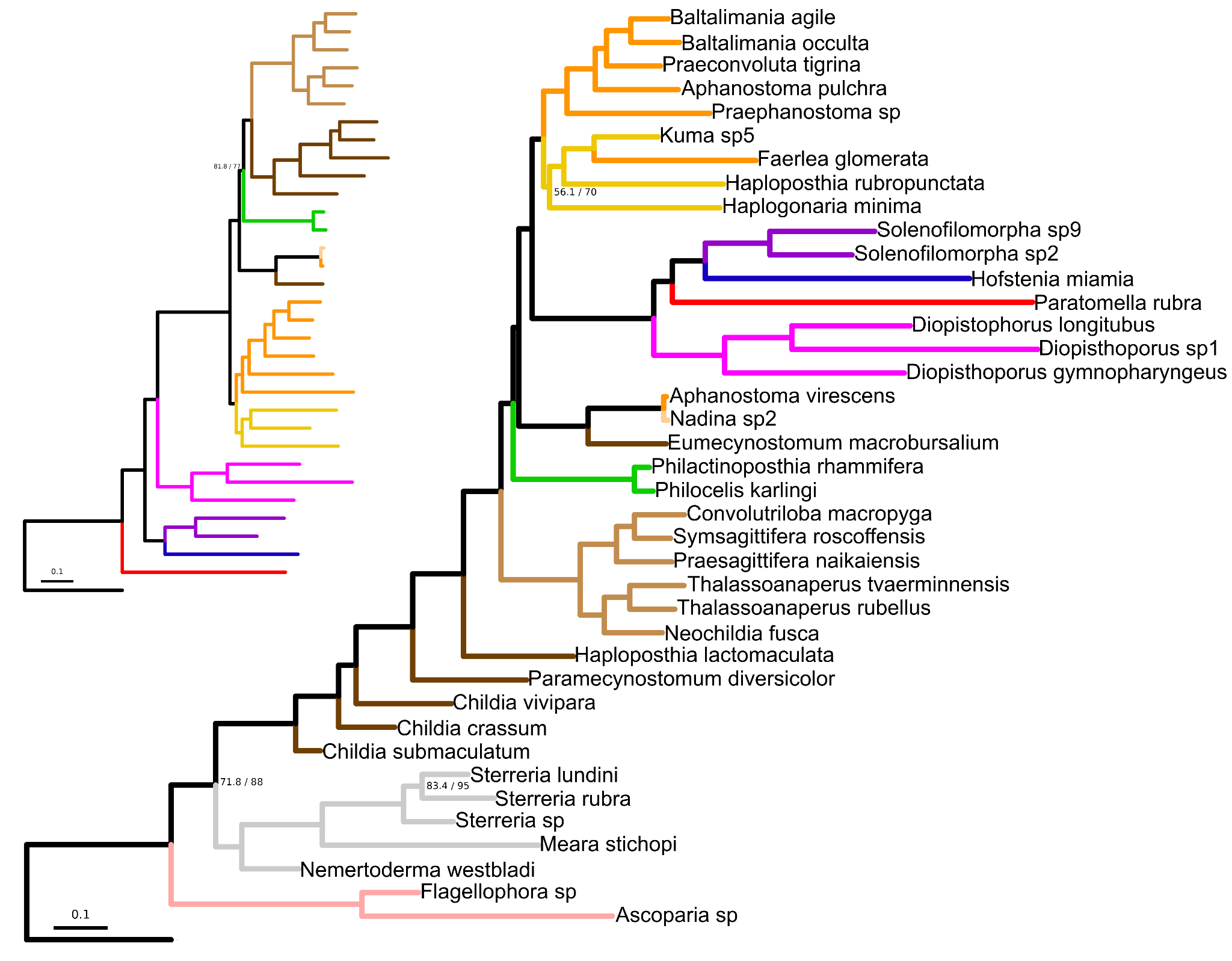


**Supplementary Figure S2** – Phylogenomic tree showcasing the difficulty of inferring a robust tree from genes filtered by average patristic distances (best 567 genes according to genesortR). We used a site-specific model with 20 amino acid categories (C20) in IQ-TREE and *Xenoturbella* as the outgroup. Unless otherwise specified, all nodes have good support (c: ultrafast bootstrap / SH-like approximate likelihood ratio test; d: posterior probabilities; >95). The scale bar indicates substitutions per site. The inset to the left shows a tree inferred from the same matrix but after removing all Nemertodermatidae species and using a typical partition model.


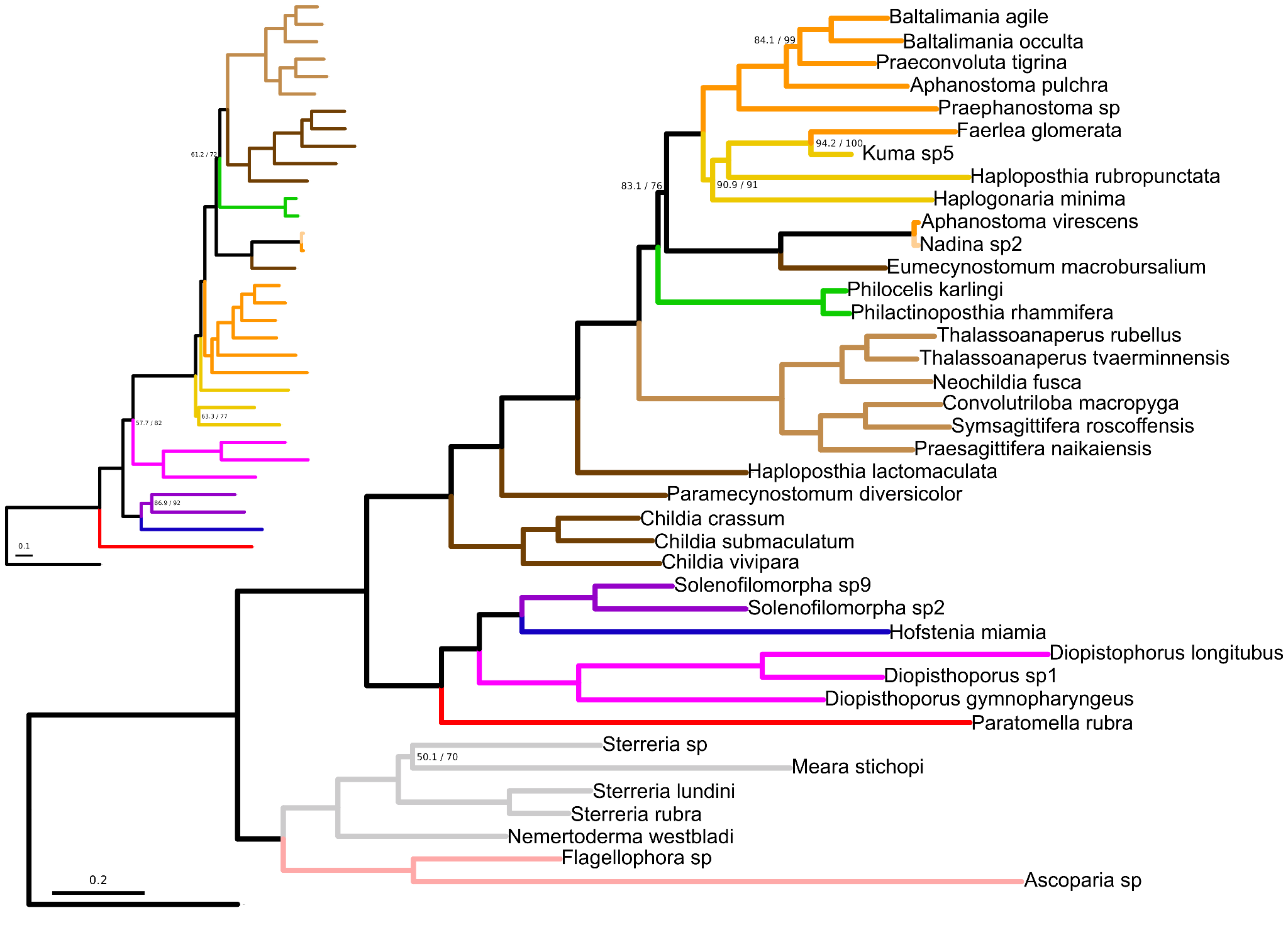


**Supplementary Figure S3** – Phylogenomic tree showcasing the difficulty of inferring a robust tree from genes filtered by compositional heterogeneity (best 567 genes according to genesortR). We used a site-specific model with 20 amino acid categories (C20) in IQ-TREE and *Xenoturbella* as the outgroup. Unless otherwise specified, all nodes have good support (c: ultrafast bootstrap / SH-like approximate likelihood ratio test; d: posterior probabilities; >95). The scale bar indicates substitutions per site. The inset to the left shows a tree inferred from the same matrix but after removing all Nemertodermatidae species and using a typical partition model.


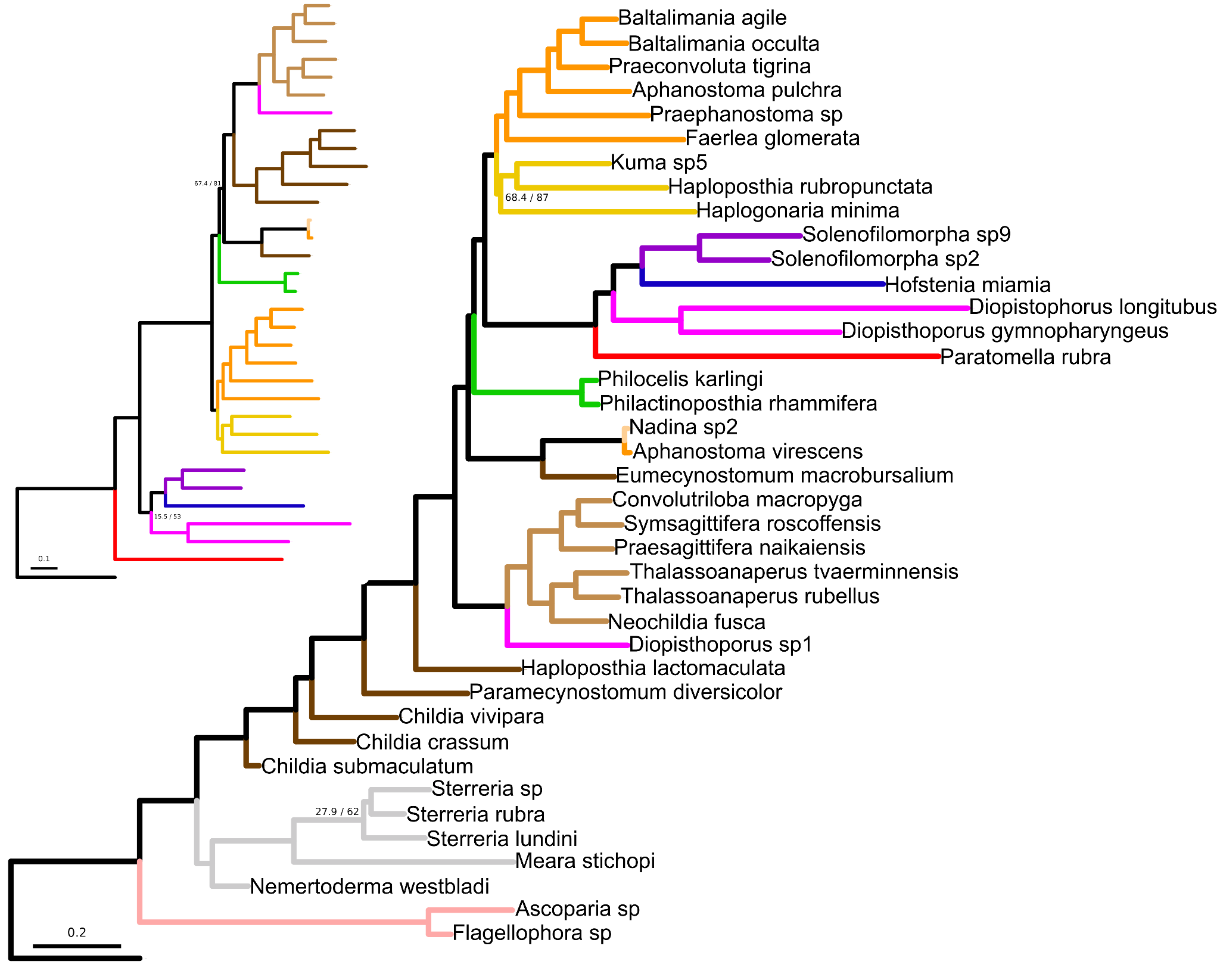


**Supplementary Figure S4** – Phylogenomic tree showcasing the difficulty of inferring a robust tree from genes filtered by saturation (best 567 genes according to genesortR). We used a site-specific model with 20 amino acid categories (C20) in IQ-TREE and *Xenoturbella* as the outgroup. Unless otherwise specified, all nodes have good support (c: ultrafast bootstrap / SH-like approximate likelihood ratio test; d: posterior probabilities; >95). The scale bar indicates substitutions per site. The inset to the left shows a tree inferred from the same matrix but after removing all Nemertodermatidae species and using a typical partition model.

**
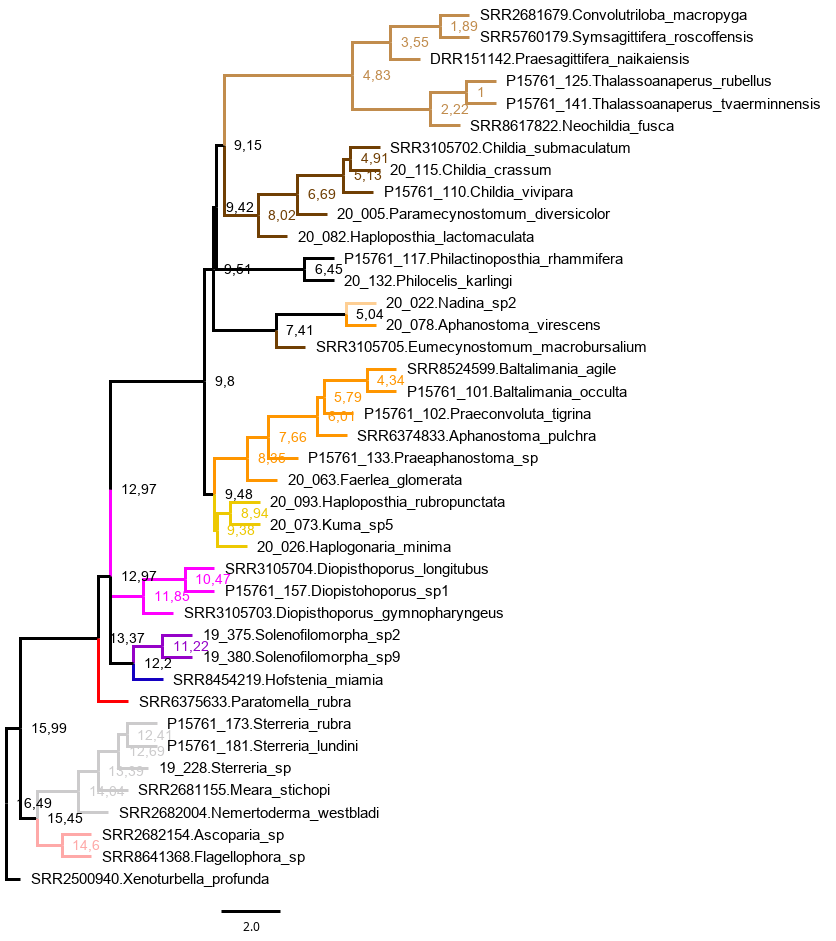
Supplementary Figure S5** – ASTRAL tree inferred from the 567 most complete genes. Branch are colour-coded as Figure 1. Nodal support represents the local posterior probability and the scale bar coalescence units. Please, note that the species *Anaperus rubellus* has been renamed to *Thalassoanaperus rubellus* during the course of this study.


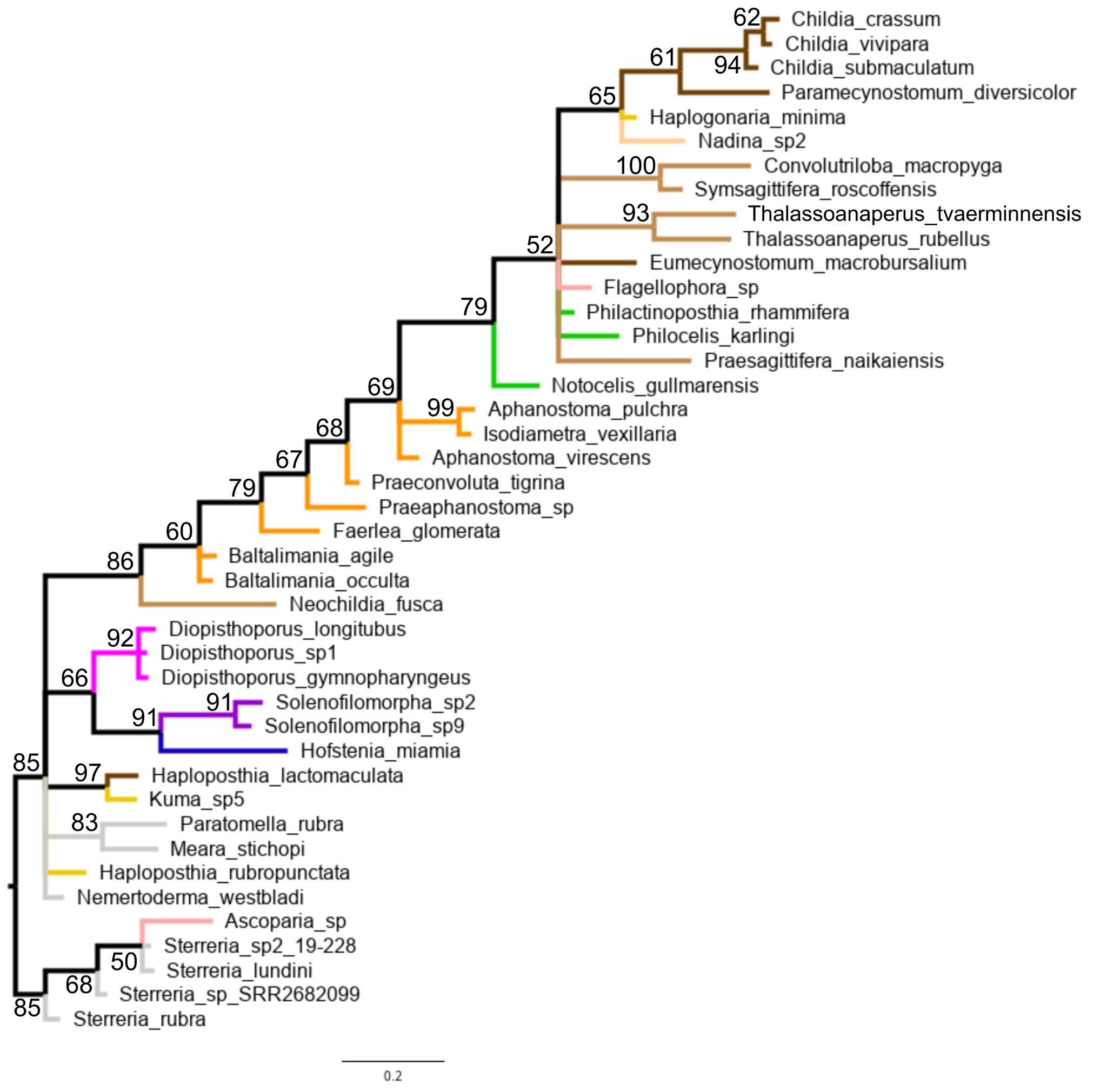


**Supplementary Figure S6** – Phylogenetic tree inferred from morphological data using MrBayes. Branch are colour-coded as Figure 1. Nodal support represents posterior probabilities and the scale bar substitutions per site.

**
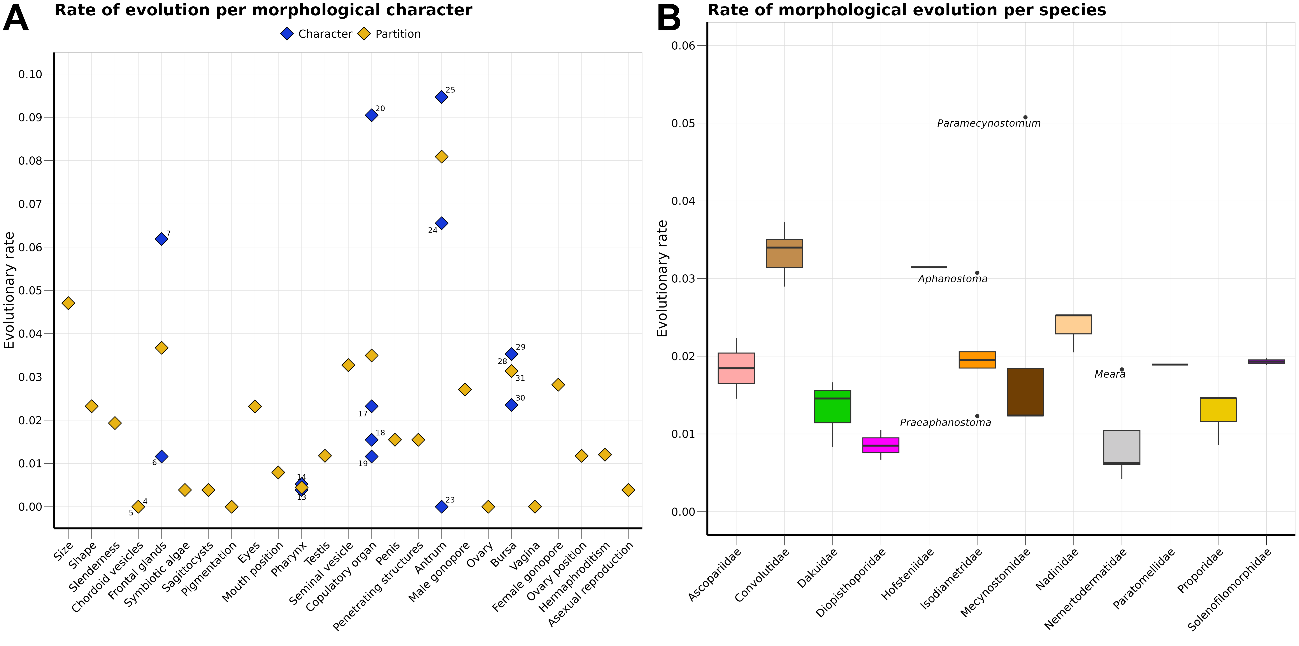
**

**Supplementary Figure S7** – Evolutionary rate inferred for each (a) character and (b) family. (a) each column represents a morphological structure, whose rate is coloured in yellow, but the individual rate of each character is also shown (in blue). (b) Boxplot calculated from the species-specific rates of each family. Boxes are colour-coded as Figure 1.


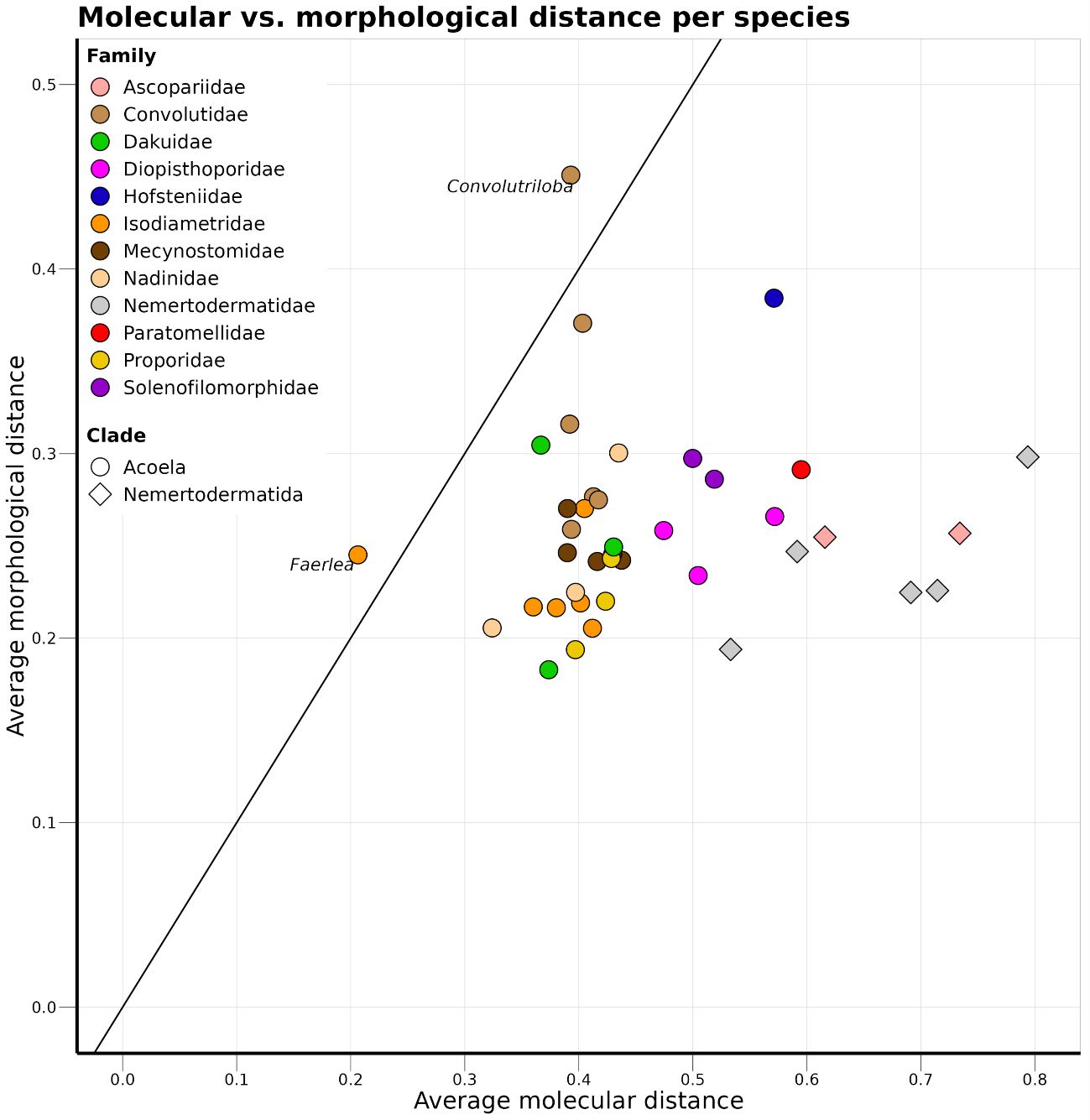


**Supplementary Figure S8** – Scatterplot of the average molecular (X-axis) and morphological (Y-axis) for each species. Dots are colour-coded as Figure 1. Nemertodermatid species are represented as diamonds and acoel species as circles.
